# Comparison of tests for spatial heterogeneity on data with global clustering patterns and outliers

**DOI:** 10.1186/1476-072X-8-55

**Published:** 2009-10-12

**Authors:** Monica C Jackson, Lan Huang, Jun Luo, Mark Hachey, Eric Feuer

**Affiliations:** 1 Department of Mathematics and Statistics, American University, Washington, DC, 20016 USA; 2 National Cancer Institute, National Institutes of Health, Rockville, MD, 20852 USA; 3 Information Management Services, Inc, Silver Spring, MD, 20904 USA

## Abstract

**Background:**

The ability to evaluate geographic heterogeneity of cancer incidence and mortality is important in cancer surveillance. Many statistical methods for evaluating global clustering and local cluster patterns are developed and have been examined by many simulation studies. However, the performance of these methods on two extreme cases (global clustering evaluation and local anomaly (outlier) detection) has not been thoroughly investigated.

**Methods:**

We compare methods for global clustering evaluation including Tango's Index, Moran's *I*, and Oden's I*_pop_; and cluster detection methods such as local Moran's *I *and SaTScan elliptic version on simulated count data that mimic global clustering patterns and outliers for cancer cases in the continental United States. We examine the power and precision of the selected methods in the purely spatial analysis. We illustrate Tango's MEET and SaTScan elliptic version on a 1987-2004 HIV and a 1950-1969 lung cancer mortality data in the United States.

**Results:**

For simulated data with outlier patterns, Tango's MEET, Moran's *I *and I*_pop _had powers less than 0.2, and SaTScan had powers around 0.97. For simulated data with global clustering patterns, Tango's MEET and I*_pop _(with 50% of total population as the maximum search window) had powers close to 1. SaTScan had powers around 0.7-0.8 and Moran's *I *has powers around 0.2-0.3. In the real data example, Tango's MEET indicated the existence of global clustering patterns in both the HIV and lung cancer mortality data. SaTScan found a large cluster for HIV mortality rates, which is consistent with the finding from Tango's MEET. SaTScan also found clusters and outliers in the lung cancer mortality data.

**Conclusion:**

SaTScan elliptic version is more efficient for outlier detection compared with the other methods evaluated in this article. Tango's MEET and Oden's I*_pop _perform best in global clustering scenarios among the selected methods. The use of SaTScan for data with global clustering patterns should be used with caution since SatScan may reveal an incorrect spatial pattern even though it has enough power to reject a null hypothesis of homogeneous relative risk. Tango's method should be used for global clustering evaluation instead of SaTScan.

## Background

In medical research and epidemiological studies, it is important to understand the spatial heterogeneity (clustering) of disease cases across the study regions. If global or local clustering patterns among the responses (e.g. cancer cases) exist, it is essential to consider the spatial correlation of the individuals in a statistical model to evaluate the association between the response (e.g., cancer death and incidence) and the risk factors. Some statistical methods that are used in spatial analysis involve aggregated summaries (count data) and Poisson regression models that assume independence of neighboring locations without overdispersion [1]. These assumptions are violated if spatial dependence exists such as in Kulldorff et al. [2] which examined unusually high breast cancer rates in the northeastern part of the United States and research done by Pickle et al. 2001 to detect a local cluster of an extreme lung cancer mortality rate in a single county in Montana caused by arsenic exposure [3]. It is important to identify models with possible spatial correlation when performing statistical analysis. For example, a risk factor (such as the distance a region is from a contaminated water source when measuring incidence of cholera) may turn out to be significant in a model assuming independent responses. However, this distance-oriented risk factor may not be identified when spatial correlation in residuals is considered in the model.

Many statistical methods for testing spatial clustering and heterogeneity were developed and can be classified into two groups: methods for global clustering evaluation and for local cluster detection [4,5]. Global clustering measures assess spatial trends (the tendency of spatial clustering) across an entire study region. Cluster detection methods identify specific local clusters. Both clustering patterns (global and local) occur in many cancer incidence and mortality data sets. Simulation studies have been conducted to evaluate methods for global clustering evaluation and local cluster detection [6-10], but none were designed to study and compare the performance of the methods on data with two extreme situations, such as global clustering and local outlier patterns. The purpose of this paper is to investigate the performance of the methods for testing spatial heterogeneity on data with the two extreme situations. We simulate disease data with homogenous populations to allow for a stronger power study [5]. We provide guidance for the proper use of the statistical methods selected in the two situations of global clustering and cluster detection.

Among methods for global clustering evaluation, we selected Tango's MEET [11], because it has been shown to be the most powerful test for testing spatial heterogeneity [5-7,12]; Moran's I [13] is selected as a reference for comparison since it has been used widely in these areas; as an alternative version of Moran's I adjusting for heterogeneous population density, Oden's I*_pop _[14] is selected because it has not been compared with Tango's MEET in earlier works. Among cluster detection methods, SaTScan elliptic version [15] is selected because it has proven to be very powerful in detecting local clusters (with either regular shapes or irregular shapes) with good precision, and reasonable computation time [16,17]. We also selected a local version of Moran's *I *among the cluster detection methods because of the ability of the statistic to perform well on outlier detection even though it is not a good method for detecting large clusters [18,19].

The rest of the paper is organized as follows. First, we briefly present selected global indices of spatial autocorrelation, local indices of spatial association (LISA) and SaTScan elliptic version. Second, we discuss the steps and parameters utilized in the simulation study. Next, the results of the simulation study are included, and the application of the selected methods on HIV and lung cancer mortality data in the United States are given. The paper ends with a discussion.

## Methods

### 2.1 Global indices of spatial autocorrelation

Global indices of spatial autocorrelation as defined by Waller and Gotway [19] provide a summary over the entire study area of the level of spatial similarity observed among neighboring observations. In this section we briefly describe the three common global indices of spatial autocorrelation we intend to evaluate. In the following sections, *i *and *j *denote geographic units (e.g. counties), *y*_*i *_is the number of cases at geographic unit *i*, *n*_*i *_is the population at risk at geographic unit *i*, , , *N *is the total number of geographic units, *w*_*ij *_is a weight assigned to the pair of geographic units *i *and *j*.

### Tango's Index

Tango's excess events test (EET) [20] is defined as

(1)

There are many choices of the weight function (see Song and Kulldorff [21] and Griffith [22]). A simple weight function based on adjacent neighbors is defined as

(2)

For this paper we refer to Tango's EET with the adjacent neighbor weight function given in equation [2] as Tango_ADJ.

We also consider a *population density adjusted exponential weight *(PD) [21] that allows for the scale of the spatial clustering to be adjusted based on the population density,

(3)

where *d*_*ij *_is the distance between geographic units *i *and *j*,  and *m*_*i *_= max{*r*:*u*_*r*(*i*) _≤ *λ*}. The population size in geographic units *i *and its *r *nearest neighbors is defined as *u*_*r*(*i*) _The parameter *λ *is chosen by the user and allows the user to view the population density as a measure of spatial clustering. Song and Kulldorff [21] note that for a given *λ*, the weight function will decrease slower in rural areas than urban areas, as in a rural area *k*_*i *_is large. Thus, a large *λ *is more sensitive to larger clustering pattern and smaller *λ *is more sensitive to smaller clusters.

Since the weight function (such as the one in equation [3]) depends on a user defined parameter *λ*, Tango developed Tango's Maximized excess events test (MEET) in order to detect clustering patterns irrespective of the geographic scale. As discussed in [11], Tango's MEET is defined as . Here, we refer to Tango's MEET in our paper as Tango_PDM since we define EET using weight as in equation [3]. We set *V *to be 50% of the total population (an upper limit on *λ*). Where *λ *has values as 0.1%, 0.5%, 1%, 2%, 5%, 10%, 20%, 30%, 40%, and 50% in our study.

### Moran's I

Moran's *I *[13] has been widely used for assessing overall clustering pattern, and is defined as

(4)

where , and .

We use the adjacent neighbors weight function as defined in equation [2]. *I *is between -1 and 1; Positive values of *I *are associated with strong geographic patterns of spatial clustering, negative values of *I *indicate negative spatial correlation (i.e. a clustering of dissimilar values), and a value close to zero represents complete spatial randomness.

### Oden's I*_pop_

Moran's *I *defined in the above paragraph does not account for population heterogeneity, therefore, a global clustering pattern indicated by a positive Moran's *I *may be completely due to the clustering of counties with similar high/low population. As alternative versions of Moran's *I*, Oden [14] derived two statistics (*I*_*pop *_and I*_pop_) to test for global spatial autocorrelation adjusting for population density. His most powerful test statistic is defined as

(5)

Where , *v*_*i *_= *n*_*i*_/*n*_+_, *v*_*j *_= *n*_*j*_/*n*_+_, *e*_*i *_= *y*_*i*_/*y*_+_, *e*_*j *_= *y*_*j*_/*y*+, and . Oden notes that symmetry is not required for I*_pop _and *w*_*ii *_≠ 0 (but can be fixed at any specified value).

For this study we refer to I*_pop _as I*_pop__ADJ with the adjacent neighbor weight function and I*_pop__PD with the population density weight function. Note that I*_pop__PD should be more sensitive to the global clustering patterns if a larger *λ *is assigned, and more sensitive to local clusters with a smaller *λ *value.

### Cluster detection methods

While global indices of spatial association evaluate the tendency of global spatial clustering across an entire region, local indices of spatial association (LISA) detect patterns in geographic units that deviate extremely from neighboring units (local outliers). SaTScan is another tool that has been widely used for local cluster detection, which is good for detecting large clusters. It may also evaluate outliers (local clusters with a small geographic or population size) when the outlier pattern is very strong or a small maximum search window is used.

### Local Moran's *I*

The local version of Moran's *I *is calculated analogously as the Moran's *I *(equation 4). Instead of computing one value of *I*_*r *_for the entire geographic region, we compute a value  for each geographic unit (e.g. county) *i*. Thus the local version of Moran's *I *is defined as



where  is the overall rate.

### SaTScan

Poisson model based SaTScan circular version [15] and SaTScan elliptic version [15] has been widely used for cluster detection on aggregated count data. For each centroid (each geographic unit is a centroid), we construct many areas (circles or ellipses) with varying sizes (and angles) sharing the same centroid. We only evaluate SaTScan elliptic version in this paper because the elliptic version has better power and precision compared with circular version for most of situations [16]. Defining the shape of ellipses by the ratio of the long radius to the short radius, we use the combination of shapes 1.5, 2, 3, and 4 in our analysis. We rotate each ellipse of fixed shape in the circle (the radius of the circle is the length of the long axis of the ellipse) sharing the same centroid, each rotation moves the ellipse to a new angle. The number of angles for those shapes 1.5, 2, 3 and 4 is selected to be 4, 6, 9, and 12, respectively. For a given zone *Z*, the likelihood is then defined as



where the *Z *is the collection of all the possible geographic units (*i*'s), with varying shapes and geographic sizes in study region, and *Z' *is the collection of geographic units in the whole study region that are not in *Z*. Also *y*_*z *_and *y*_*z *_are the numbers of cases inside and outside of *Z*, respectively, and *n*_*z *_and *n*_*z *_are the corresponding populations. When we are interested in clusters of both high and low rates, the indicator can be removed. The statistic is then



where *G *is the whole study region and



which is independent of the *Z*. The *Z *that maximizes the *λ *over all the Z's in G is the most likely cluster. When searching for large clusters, we use 50% of total population as the maximum size of *Z*, and for local small cluster or outlier detection, we use 5% of the total population as the maximum size of *Z *in the simulation.

### Simulating geographic data

We simulate count data with fixed total number of cases (or sample size) for *i *= 1,...,3109 representing data from the 3109 counties of the continental United States (US) (multinomial data.) All distance measures are calculated using the latitude and longitude coordinates of the county centroids. We use the actual configuration of U.S. counties but not the real U.S. populations for the counties, because the U.S. has a complex configuration at the county level with a varying number of neighbors for each county and it can provide a real and complicated structure of the adjacent neighbors and nearest neighbors. Waller et al. [5] discussed the issue with power analysis of the tests of clusters and clustering in heterogeneous populations. They found that power depends on the local population at risk. Here, in order to evaluate the performance of the methods on data with varying relative risk patterns without confounding from heterogeneous population, we simulate data assuming homogeneous county population in the US. We set *n*_*i *_(the population at risk) equal to 5,000 for all simulations, thus *n*_+ _= 15,545,000. Note that the performance of the tests will be poorer when data are generated from a heterogeneous population, because more spatial variation will be introduced through the population variation. We experimented with simulations that had 5,000-50,000 fixed cases (results not shown) to obtain a relevant range of power. Larger and smaller number of cases yielded results which did not adequately force separation of the methods with respect to the power in different scenarios (i.e., for small number of cases all methods had a very poor power for data with outliers; and for a larger number of cases, all the methods had a power approximately 1 for data with a global clustering pattern.) Therefore, we used a smaller *y*_+ _(sample size of 5,000) to simulate data with global clustering pattern and a larger *y*_+ _(sample size of 30,000) to simulate data with a local cluster (outlier) as described in the following sections.

We simulate regional count data sets under the null hypothesis of constant relative risk

(6)

where (*y*_1_, *y*_2_,...,*y*_3109_) are regional counts generated from the multinomial distribution. The total number of cases *y*_+ _here is always the same as the total number of cases in the corresponding data simulated under the alternative hypothesis.

We simulate regional count data sets under the alternative hypothesis

(7)

where , *r*_*i *_is the relative risk at geographic unit *i*, which is not the same for all geographic units.

**Figure 1:**
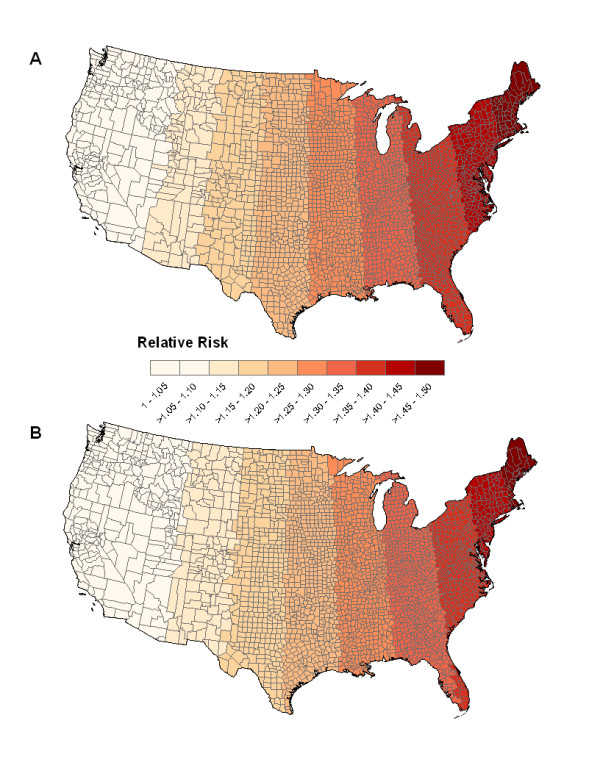
**Global pattern**. Graphical representation of simulated data with global clustering patterns. The risk increases from West to East with either an exponential monotone function (A) or a linear monotone function (B).

In order to simulate data with global clustering pattern, the relative risks are defined to have a directional west-east trend with either a linear or exponential increasing function (this trend can also reflect the pattern in data with a north-south trend since a simple rotation is all that is necessary.) With the minimum relative risk (on the west) as 1, we experimented with maximum relative risk (on the east) with values ranging from 1.5-10 (results not shown). However, we were unable to detect differences in the performance of the selected statistics when the relative risk was above 2 (all methods had power close to 1). Therefore we used a maximum relative risk of 1.5 in our final study. The monotone increasing function is defined as

(8)

for the relative risks with a linear trend, and

(9)

for the relative risks with an exponential trend, where *a *is an integer from 0-99. The longitudinal coordinates of the continental United States ranges from -124.161 to -67.623. We divide this large interval into 100 sub-intervals of equal length where each interval corresponds to a value of *i *used in equations [8] and [9]. Both equations allow for a relative risk of 1.5 between the lowest and highest longitudinal coordinates. As shown in Figure 1, the relative risk increases from west to east monotonically, while the relative risk with the exponential trend (figure 1A) increases faster than that with the linear trend (figure 1B).

**Figure 2:**
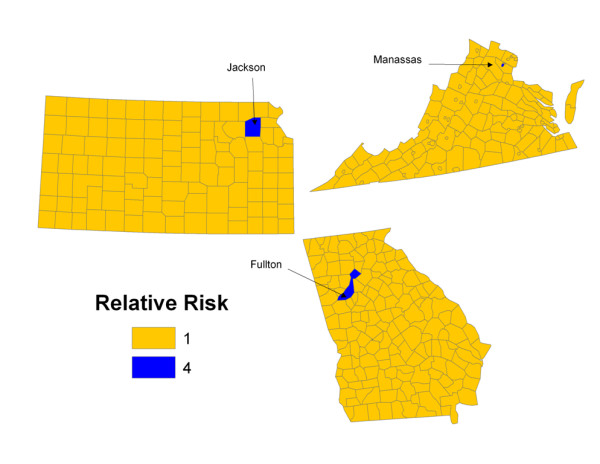
**Local cluster**. Graphical representation of the simulated relative risk for each of the three alternative data set with local clustering. The relative risk for the counties of Manassas City Virginia (2 adjacent neighbors), Jackson County Kansas (6 adjacent neighbors), and Fulton County Georgia (10 adjacent neighbors) are set to 4. All other counties in the United States (excluding Alaska and Hawaii) are set to 1.

In order to simulate data with small local clusters (outlier), we set all values of *r*_*i *_equal to 1, except for one geographic unit which has a relative risk of 4. We only consider a single outlier in the simulated data. The number of adjacent neighbors for all counties in the continental U.S. ranges from 0-14. In order to evaluate if the power varies by the number of adjacent neighbors around one outlier, we select three counties with different number of adjacent neighbors as the outliers with relative risk of 4, which are Manassas City, Virginia (with 2 adjacent neighbors), Jackson County, Kansas (with 6 adjacent neighbors), and Fulton County, Georgia (with 10 adjacent neighbors). Figure 2 includes the maps of the assigned relative risks for the three patterns.

### Calculating power and chance of a county being detected as inside clusters

We use Monte Carlo simulation methods to assess the power of rejecting null hypothesis and the chance a county is detected as inside any cluster. The steps involved in our simulation study are as follows:

• Step 1: Simulate 10,000 data sets using the multinomial distribution (equation 6) under the null hypothesis (no outlier or global clustering pattern).

• Step 2: Calculate Tango_PDM, Tango_ADJ, Moran's *I*, I*_pop__PD, I*_pop__ADJ, and SaTScan elliptic version (SaTScan-E) statistics for each data set from step 1. Calculate Local Moran's I for each county *i *in each data from step 1.

• Step 3: Find the 95th percentile for Tango_ADJ, Moran's *I*, I*_pop__PD, I*_pop__ADJ, and SaTScan-E. The 95^th ^percentile is the *critical point *for the empirical distribution of each of the statistics. Find the 5th percentile for Tango_PDM, which serves as the *critical point *for Tango's *I *with the PDM weight function. Find the 95th percentile for the local Moran's *I *at each county *i*. There are then 3109 critical points for Local Moran's *I *(one for each county.)

• Step 4: Simulate 1000 alternative data sets using the multinomial distribution (equation 7) under each of the alternative hypotheses described in the previous section. The five settings mentioned above are:

a) Global pattern with exponentially increasing relative risk rates (equation 9)

b) Global pattern with linearly increasing relative risk rates (equation 8)

c) Local outlier with two adjacent neighbors (Manassas City VA)

d) Local outlier with six adjacent neighbors (Jackson County KA)

e) Local outlier with ten adjacent neighbors (Fulton County GA)

• Step 5: Calculate Tango_PDM, Tango_ADJ, Moran's *I*, I*_pop__PD, I*_pop __ADJ, and SaTScan-E for each of the 1000 data sets from Step 4. Calculate local Moran's *I *for each county in each data set from Step 4.

• Step 6: Report the power depending on the statistics.

a. For each of the statistics Tango_ADJ, Moran's *I*, I*_pop__PD, I*_pop__ ADJ, and SaTScan-E, We calculate the power as the percentage of values out of the 1000 replicates that are above the critical point obtained in step 3. For Tango_PDM, the power is the percentage of values smaller than the critical point obtained in step 3

b. For the cluster detection methods SaTScan-E and local Moran's *I*, we also report the chance of a county being detected inside clusters, which is defined to be the number of times that a county is counted inside a detected cluster or as an outlier out of the 1000 replicates. If that ratio for a county is close to 1, then there is a large chance that a particular county is inside a cluster or an outlier as determined by this method. If the ratios for the counties inside the true cluster or a true outlier are all high, we claim that the method has a high chance to find the correct cluster location, which indicates good precision of cluster detection.

### Notes on simulation methods

Table 1 includes a summary of all the statistics and weight functions we use for this study. Since the adjacent neighbor weight function is one of the most common weight functions used in spatial data analysis we included it in our study. For comparison we also used a population density weight function which utilized the population when determining weights. Methods for global clustering evaluation cannot detect local cluster locations; therefore we do not evaluate the chance of a county being detected inside a cluster as defined in step 6c for those methods. Note that we evaluate the performance of Moran's *I*, Tango's statistics, Oden's I*_pop _and SaTScan-E for data with global clustering pattern and outlier pattern. We report statistical power for all methods except local Moran's *I*. Local Moran's *I *is only used for local outlier identification and we only report chance of geographic unit being detected as an outlier in the outlier detection for Local Moran's *I *since a power calculation is not possible due to fact that Local Moran's *I *provides a statistic for each geographic unit. For SatScan-E we report both power and the chance of geographic units being detected as inside clusters. The results of the chance of being inside clusters are presented in maps and the powers are provided in a table.

**Table 1:** Statistics and methods used to detect global clustering and outlier detection along with weight functions used.

		**Weight function**	
	**Method/Statistic**	**Adjacent neighbor**	**Population density**

**Global**	Tango	✓	✓
	I* _*pop*_	✓	✓
	Moran's I	✓	

**Local**	Local Moran's I	✓	
	SatScan-E	na	na

## Simulation results

### Performance of the methods on data with global spatial clustering patterns

Among all the selected methods for global clustering evaluation, Tango_PDM has the highest power to reject the null hypothesis of homogeneous relative risk over the entire study region and claim that the simulated data have global clustering pattern (results presented in Table 2.) The I*_pop _statistics with varying weights also has good power (>0.8) for the population density weight function. Specifically, when there is a large *λ *(50% of total population) in the population density weight function, the statistics have good power because they are more sensitive to large clustering pattern according to the formulation. All methods using an adjacent neighbor weight functions (Tango_ADJ, Moran's *I*, I*_pop__ADJ) that only evaluate limited adjacent neighbor regions have low power (around 0.1-0.2) for detecting the global clustering patterns.

**Table 2:** Power of selected statistics for detecting data with local and global cluster types.

		**Global clustering methods**						**Local cluster detection methods**	
			
		**Tango_PDM**	**Tango_ADJ**	**Moran's *I *ADJ**	I*_pop_ _PD **(*λ *= 5%)**	I*_pop_ _PD **(*λ *= 50%)**	I*_pop_ _ADJ	**Local Moran's *I***	**SaTScan^a^**
**Local clusters** relative risk = 4 sample size = 30,000	Two adjacent neighbors (Manassas City VA)	0.107	0.127	0.024	.079	0.056	0.060	+	0.973
	Six adjacent neighbors (Jackson County KS)	0.089	0.124	0.027	.087	0.042	0.047	+	0.962
	Ten adjacent neighbors (Fulton County GA)	0.081	0.125	0.030	.079	0.043	0.055	+	0.979

**Global clustering** relative risk = 1.5 sample size = 5.000	Linear	0.997	0.285	0.232	.884	0.996	0.119	+	0.737
	Exp	0.998	0.291	0.235	.886	0.997	0.129	+	0.781

^a ^for global clustering evaluation, we used 50% of total population as the maximum search window. With the outlier detection, we use 5% of the total population as the maximum search window in SaTScan-E.

+ Power calculations are not available for Local Moran's *I *due to the fact that a statistics is produced for each geographic unit in the study. It is difficult to define power in the presence of multiple comparisons.

Designed for cluster detection, SaTScan-E with maximum spatial window as 50% of total population has moderate power (around 0.75 (See Table 2)) in identifying the data with spatial heterogeneity successfully.

The type of increasing function in the global trend (exponential or linear) was not a key factor in power for all the statistics for the data with the same maximum relative risk. Power is slightly higher for data with exponential function compared with linear function because the relative risk in data with exponential function increases faster than that in data with linear function, even though the maximum relative risk in both types are the same (maximum relative risk = 1.5). We also note that with maximum relative risk above two, all methods obtained a statistical power above .95.

**Figure 3:**
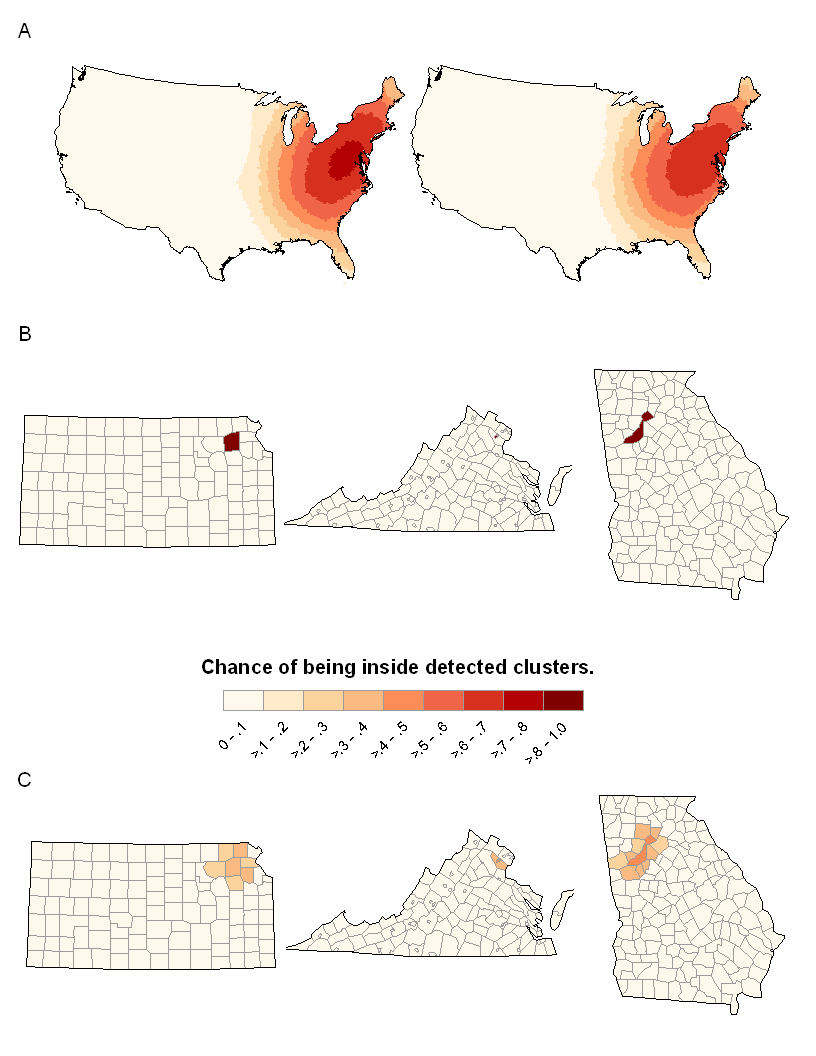
**SatScan and local version of Moran's I results for Global clustering and local cluster patterns**. Graphical representation showing the chance of geographic units being detected as inside clusters (the number of times a geographic unit was selected as a unit inside a cluster over 1000 simulations). (A) Results from SatScan-E on data with global clustering pattern as shown in Figure 1, (B) Results from SatScan-E on data with outlier as the three counties shown in Figure 1, and (C) Using local version of Moran's I on data with outlier pattern as shown in Figure 2.

SaTScan-E also detects cluster locations with significantly higher risks. We compute the chance of a county being inside the detected clusters out of the 1000 replicates and present the chances in Figure 3A. The counties with orange through red color have moderate to high chance (60% to 100%) being detected as inside clusters of high risk. The blue color areas indicate counties with low chance to be detected. The patterns in Figure 3A do reflect a spatial variation (high risk in the west vs. low risk in the east). The chances of counties being inside detected clusters are lower for data with linearly increasing function (right) compared with that with exponentially increasing function (left), which is expected because the risks increase faster with an exponential function. Compared with the true risk pattern in Figure 1, SaTScan-E only detects clusters with high risk in limited eastern areas, but does not capture the global clustering pattern as shown in Figure 1. Also, we cannot evaluate the precision of SaTScan-E because there is no true cluster in the data with global clustering pattern.

### Performance of methods for data with local clusters (outliers)

Based on our simulation study, the ability of the statistics to identify the spatial heterogeneity in data with only outliers is not very sensitive to the number of adjacent neighbors. SatScan-E with maximum search window as 5% of total population performed significantly better (with power above 0.95) than all methods for global clustering evaluation (with power lower than 0.2) in terms of power (Table 2). This result was not surprising since SaTScan-E is designed for local cluster detection and with a small search window it searches for clusters with a smaller size (outliers). Methods for global clustering evaluation do not perform well because there is no strong global clustering tendency when the data have a single outlier including one county.

We also compared the chance of the true outlier counties being detected as inside clusters from the two methods for local cluster detection (SaTScan-E vs. LISA) in Figure (3B vs. 3C). SaTScan-E has a very good chances (above 0.9) to detect the true outlier counties. The maximum chance of being detected as inside clusters for local Moran's I are lower than those from SaTScan-E (around 0.4-0.5). However, we note that local Moran's *I *did detect the location of the true simulated outlier with a higher chance (0.4-0.5) than all other counties (with chance < 0.1) as shown in Figure 3C. The precision for SaTScan-E is very good because only the true outlier counties have a very high chance (red color) to be detected from the map, and the precision of local Moran's *I *is not very good because the chance of a true outlier being detected is about 0.4-0.5 and the counties around the true outliers also have moderate chance (0.4-0.5) to be detected as inside cluster.

## Examples

We provide two real data examples with the two extreme clustering situations (global clustering pattern and outlier) to demonstrate the performance of SaTScan-E and Tango_PDM, which have the best performance among the others methods evaluated in our simulation study.

### Clustering pattern of HIV mortality in US during recent decades

**Figure 4:**
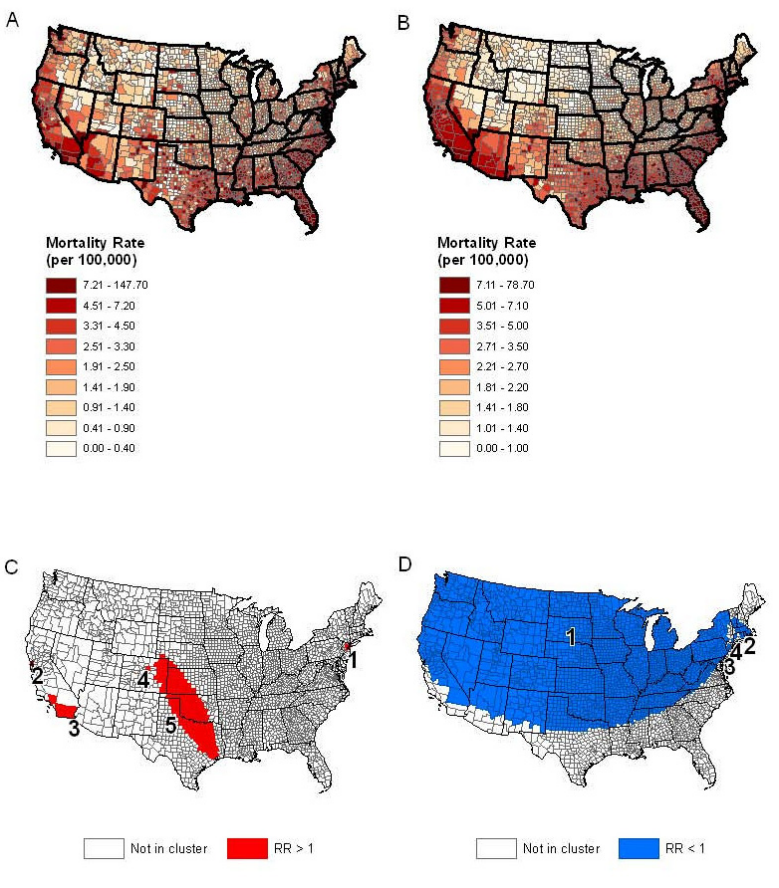
**Example using data with a global clustering pattern**. (A) Raw and (B) Smoothed HIV mortality rates per 100,000 for the years 1987-2004 using county population as weight for smoothing over 30 nearest neighbors (top). (C) is detected clusters of high rates from SaTScan-E and (D) is detected clusters of low rates from SaTScan-E

HIV disease, as a non-cancer disease, has the public's attention in recent decades because of its high mortality. To understand the spatial pattern of HIV mortality, we use an HIV mortality data during 1987-2004, which is available from the National Center for Health Statistics and extracted from the Surveillance, Epidemiology, and End Results (SEER) Program of the National Cancer Institute (NCI) . The total number of HIV deaths during 1987-2004 is 406,465 in our data set. First, we simply map the observed mortality rate and the smoothed rate in Figure 4A (observed) and 4B (smoothed) using GIS. In Figure 4B, the rates are smoothed using the software Headbang [23] with the county population at risk as the weight for smoothing with 30 neighboring counties. The smoothed map provides a better sense of spatial pattern with less noise compared with the observed map. From the maps, we visually see a south to north global clustering trend of the mortality rates similar to the global clustering trend we evaluated in our simulation study except in a different direction (West to East trend in our simulated data set.)

We then used the selected methods on the raw HIV mortality data for testing of the spatial heterogeneity. Tango_PDM successfully detected the global clustering pattern (p-value < .001). This result from Tango_PDM indicates that it is necessary to consider global spatial correlation of mortality rates when constructing classical spatial statistical model to analysis mortality data. A spatial model with a variance-covariance matrix incorporating spatial patterns will help to eliminate a possible correlation in the residuals compared with a regression model without considering spatial correlation.

As shown in Figure 4C, SaTScan-E identified five statistically significant clusters (p-value < .05) with relative risks ranging from (1.11 to 10.37) when we only searched for clusters of high rates using 50% of total population at risk as the maximum spatial search window. However, it does not capture the global clustering pattern observed in Figure 4A and Figure 4B. We also conduct a search for clusters with low rates only. As shown in Figure 4D, we have a large cluster (No. 1) covering the upper part of US with low relative risk (0.31) compared with the regions outside. The other significant clusters (No. 2, 3, and 4) have relative risks as 0.52, 0.53 and 0.67 separately. This does indicate a global clustering pattern in HIV mortality in US, which is consistent with the pattern observed in Figure 4A and Figure 4B.

### Clustering pattern of lung cancer mortality in US during 50s-60s

Lung cancer has been the leading cancer in the United States and experienced a decreasing mortality trend over years. The mortality is very high for lung cancer patients in earlier years (1950s-60s). Here, our interest is to identify possible clusters/outliers with high lung cancer mortality or global clustering pattern using the two selected methods. We obtained the lung cancer mortality data during the years 1950-1969 for white males from the National Cancer Institute at . The total number of deaths observed in these data are 570,521 (a large sample size). When Tango_PDM was used, we obtained a p-value <.001, which indicates the existence of a global clustering pattern in this male lung cancer data.

**Figure 5:**
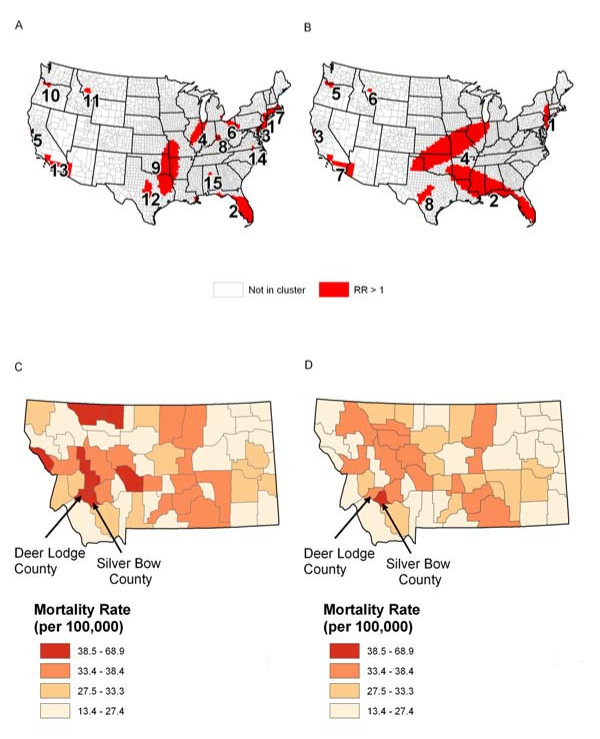
**Example using data with a local cluster pattern**. (A) is detected clusters of high rates from SaTScan-E with 5% maximum search window and (B) is detected clusters of high rates from SaTScan-E with 50% maximum search window. (C) Raw and (D) Smoothed lung mortality rates per 100,000 for the years 1950-69 using county population as weight for smoothing over 30 nearest neighbors.

SatScan-E with maximum search window as 5% of total population at risk detects 15 significant clusters of high rates with p-values smaller than 0.05 (5A). When 50% of total population at risk is used, 8 clusters of high rates are detected with p-values smaller than 0.05 (5B). The relative risks in those detected clusters are ranging from 1.07 to 1.81. There are many clusters with only one or several counties (outliers) in both maps and there are more outliers detected in Figure 5A. We notice a small cluster including only two counties (Silver Bow County and Deer Lodge County) in the state of Montana, which is a typical type of outlier with high relative risk (1.81). The mortality rate inside this outlier region is 67.1 per 100,000 people vs. 37 per 100,000 outside. The observed mortality rates in Montana are presented in Figure 5C and the smoothed rates (using Headbang software) is in Figure 5D, which also shows a possible outlier location in Silver Bow county and Deer Lodge County. It has been known that Silver Bow County was the location of the pollution source caused by a copper plant which explained the high risks of lung cancer mortality in this area. This is also consistent with the results noticed by Lee et al. [3] that an extreme lung cancer mortality rate in a single county (Silver Bow County) in Montana was caused by arsenic exposure. Here, we can claim that there exist both global clustering pattern and outliers in white male lung cancer mortality during 1950s-60s in this county.

## Conclusion and discussion

In this article, we explored the performance of several global indices of spatial autocorrelation, local Moran's *I *and SaTScan-E to detect global clustering and identify outliers. Tango_PDM had the highest statistical power for identifying global clustering patterns among all methods considered. The power for I*_pop _with proper *λ *are very close to that for Tango_PDM, but the user has to choose the value of *λ*, which is subjective and it is very hard to find the optimal *λ*. However, Tango_PDM evaluates regions with varying total population and provides a single statistic and p-value, so it is easier for users without much knowledge of the geographic features of the study region to identify global spatial clustering. SaTScan with a large search window (50% of total population) may have moderate power to detect spatial heterogeneity but it may not reveal the correct global clustering pattern.

SaTScan-E performed well in detecting the outliers in terms of power, which is much better than local Moran's *I *and the methods for global clustering evaluation. From our simulation study we also found that for a large relative risk difference (greater than 2), SaTScan-E as well as all the other methods considered were able to detect spatial heterogeneity with a power above .98. For local cluster detections (outlier), the power is sensitive to the change in sample size with increased sensitivity. When the sample size was less than 25,000 we had very low power in detecting outliers. Even though SaTScan and local Moran's *I *did obtain a higher chance to locate the true location of outliers compared with other locations, the overall chance of detecting the true outlier is low.

The weight function does affect the power of the methods for evaluating global clustering pattern. For the methods using adjacent neighbor weight function, the order of the power is Tango's Index, I*_pop_, and Moran's *I*, which implies that Tango's index is the best in detecting spatial heterogeneity among the selected methods for global clustering evaluation even with the same weight function. When comparing the power for I*_pop _with the weight functions PD (with *λ *= 50% of total population) and ADJ, we notice that the power for the data with global clustering is much higher with the PD weight function compared to the ADJ weight function.

A limitation of our simulation is that we simulated homogeneous county populations across an entire region. This assumption likely does not hold in real data. Heterogeneous population densities complicate comparisons of statistical power between hypothesis test evaluating spatial clusters or global clustering [5]. Our intent was to investigate statistical power while controlling for as many variations and confounding factors as possible, in order to effectively evaluate and compare each statistics. However, we do provide an example based on real mortality data with heterogeneous population across the U.S.

Our example based on heterogeneous population revealed that Tango_PDM method detected the global clustering pattern which validates the simulation results based on a similar global clustering pattern. Although SaTScan was designed for cluster detection, it can still be used to access global clustering patterns if larger clusters exist. However, SaTScan can be misleading in detecting clusters as shown in Figure 4c. Therefore one should be careful when applying SaTScan to evaluate global clustering patterns. SaTScan did find the outlier (Silver County) in Montana with real heterogeneous population and unusually high lung cancer mortality rate among white males using a 5% and 50% window. Usually, it is difficult to detect outliers using a 50% window. In our case a 50% window was sufficient to detect an outlier. This result is partially due to the fact that we have a large relative risk of 1.8 and a large sample size which made outlier detection more feasible.

There are many directions for future research. First there are several alternative autocorrelation and spatial association patterns (not only global clustering and outlier patterns) that could be considered. For example, Song and Kulldorff [7] simulate various cluster sizes for their power study. In addition, there is much work to be done in defining appropriate spatial weight functions. We considered common spatial weights: adjacent neighbors and population density. However alternative weight functions (such as the weight functions included in Song and Kulldorf [21]) may influence the performance of each test. Other outlier detection methods (e.g. Multi-item Gamma Poisson Shrinker [24] (MGPS)) may be evaluated and compared with SaTScan-E and Local Moran's *I *in the future. Comparison with heterogeneous population data is also possible if one is interested in the performance of the tests in the presence of real heterogeneous population from the US.

## Competing interests

The authors declare that they have no competing interests.

## Authors' contributions

MCJ, LH and EF conceived and designed the study, interpreted the simulation results, and prepared the manuscript. MH and JL wrote the simulation routines. All authors read and approved the final manuscript.
